# Hippocampal Impairment Triggered by Long-Term Lead Exposure from Adolescence to Adulthood in Rats: Insights from Molecular to Functional Levels

**DOI:** 10.3390/ijms21186937

**Published:** 2020-09-21

**Authors:** Ana Carolina Alves Oliveira, Aline Dionizio, Francisco Bruno Teixeira, Leonardo Oliveira Bittencourt, Giza Hellen Nonato Miranda, Géssica Oliveira Lopes, Everton L. P. Varela, Mariane Nabiça, Paula Ribera, Kelly Dantas, Aline Leite, Marília Afonso Rabelo Buzalaf, Marta Chagas Monteiro, Cristiane Socorro Ferraz Maia, Rafael Rodrigues Lima

**Affiliations:** 1 Laboratory of Functional and Structural Biology, Institute of Biological Sciences, Federal University of Pará, Belém, PA 66075-110, Brazil; anacarolina@ufpa.br (A.C.A.O.); teixeirafb@ufpa.br (F.B.T.); leo.bittencourt25@gmail.com (L.O.B.); gizahellen@hotmail.com (G.H.N.M.); gessicalopes_22@hotmail.com (G.O.L.); 2 Department of Biological Sciences, Bauru School of Dentistry, University of São Paulo, Sao Paulo 17012-901, Brazil; alinesdionizio@usp.br (A.D.); lima.gbm@gmail.com (A.L.); mbuzalaf@fob.usp.br (M.A.R.B.); 3 Laboratory of Clinical Immunology and Oxidative Stress, Pharmacy Faculty, Institute of Health Science, Federal University of Pará, Belém, PA 66075-110, Brazil; evertonlpvarela@gmail.com (E.L.P.V.); martachagas2@yahoo.com.br (M.C.M.); 4 Laboratory of Applied Analytical Spectometry, Institute of Exact and Natural Sciences, Federal University of Pará, Belém, PA 66075-110, Brazil; mariane_gama@hotmail.com (M.N.); kdgfernandes@ufpa.br (K.D.); 5 Laboratory of Inflammation and Behavior Pharmacology, Pharmacy Faculty, Institute of Health Science, Federal University of Pará, Belém, PA 66075-110, Brazil; paularibera17@gmail.com (P.R.); crismaia@ufpa.br (C.S.F.M.)

**Keywords:** lead, long term exposure, hippocampus, memory

## Abstract

Lead (Pb) is an environmental and occupational neurotoxicant after long-term exposure. This study aimed to investigate the effects of systemic Pb exposure in rats from adolescence to adulthood, evaluating molecular, morphologic and functional aspects of hippocampus. For this, male Wistar rats were exposed to 50 mg/kg of Pb acetate or distilled water for 55 days by intragastric gavage. For the evaluation of short-term and long-term memories, object recognition and step-down inhibitory avoidance tests were performed. At the end of the behavioral tests, the animals were euthanized and the hippocampus dissected and processed to the evaluation of: Pb content levels in hippocampal parenchyma; Trolox equivalent antioxidant capacity (TEAC), glutathione (GSH) and malondialdehyde (MDA) levels as parameters of oxidative stress and antioxidant status; global proteomic profile and neuronal degeneration by anti-NeuN immunohistochemistry analysis. Our results show the increase of Pb levels in the hippocampus of adult rats exposed from adolescence, increased MDA and GSH levels, modulation of proteins related to neural structure and physiology and reduced density of neurons, hence a poor cognitive performance on short and long-term memories. Then, the long-term exposure to Pb in this period of life may impair several biologic organizational levels of the hippocampal structure associated with functional damages.

## 1. Introduction

Lead (Pb) is a metal found in the earth’s crust, occurring in deposits mainly combined with other elements, as galena (PbS), a Pb sulfide commonly associated with silver. Its presence in the atmosphere occurs mostly from human activities, representing a serious environmental problem once it is not a biodegradable element and has a bioaccumulative neurotoxic characteristic in humans [1].

The World Health Organization classifies Pb as one of the chemical elements that offer the greatest risk to human health, being able to reach and create deposits in different tissues and organs [2]. It has been widely proposed and accepted that the central nervous system (CNS) is the main target after Pb exposure or poisoning, highlighting that the developing brain presenting itself as particularly sensitive to these effects [3,4,5]. Interestingly, little has been described about the effects of Pb on the CNS in adolescence or adulthood, especially on the hippocampus [6,7].

Anatomically, the hippocampus is a brain structure located deep in the medial temporal lobe, being responsible for several cognitive functions, as learning and memory processes [8], storage and processing of spatial functions [9] formation and storage of episodic memory [10], working memory [11] and spatial memory [12,13].

The aim of this work was to investigate in adult rats the effects of systemic exposure to Pb, from adolescence to adulthood, on the functioning and hippocampal structure. Our hypothesis is that exposure to Pb in this period of life is capable of triggering biochemical and proteomic changes associated with hippocampal neurodegeneration, culminating in impaired memory and learning.

## 2. Results

### 2.1. Long-Term Exposure to Pb Promotes Short-Term Memory Impairment in the Object Recognition Task

**Figure 1:**
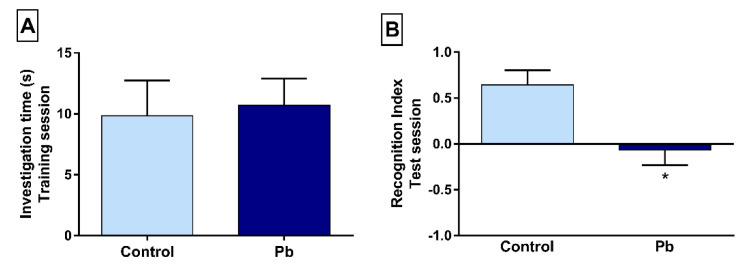
Effects of long-term exposure to Pb on short-term memory, evaluated by object recognition test. (**A**) Columns represent the total investigation time of object C1 plus the investigation time of object C2 (C1 + C2), counted in seconds; (**B**) the recognition index was calculated by the time the animals investigated the new and familiar objects (T - C3/ T + C3) during the test phase, performed 30 min after the training. The results are expressed in mean ± S.E.M of the investigation time in the training phase and recognition index in the test phase, for 3 min. * *p* < 0.05 compared to the control group (Student’s *t*-test).

The behavioral evaluation of rats exposed to Pb or H_2_O_dest._ on the object recognition shows that both experimental groups explored equally the similar objects in the training session (control: 9.85 ± 2.89; Pb: 10.71 ± 2.17; *p* > 0.05; Figure 1A). In the short-memory evaluation, performed 30 min later after training phase (Figure 1B), animals exposed to Pb showed poor performance according to the recognition index (control: 0.64 ± 0.15; Pb: −0.06 ± 0.16; *p* = 0.003), which reflects poorly short-term memory skill.

### 2.2. Long-Term Exposure to Pb Causes Long-Term Memory Impairment

**Figure 2:**
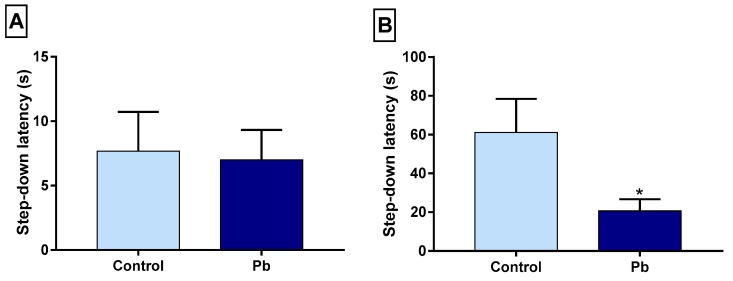
Effects of long-term exposure to Pb on step-down inhibitory avoidance. (**A**) step-down latency time (s) in the training session; (**B**) step-down latency time 24 h later. * *p* < 0.05 compared to the control group (Student’s *t*-test).

To assess the long-term memory, we employed the inhibitory avoidance task. As previously achieve in the short-term evaluation, both tested groups performed the training session, similarly, descending the safe platform (Figure 2A). Twenty-four hours after the training session, long-term exposure to Pb displayed a reduction in the step-down latency parameter (control: 61.11 ± 17.38; Pb: 20.73 ± 6.02; *p* = 0.028), which suggests long-term memory deficit (Figure 2B).

### 2.3. Exposure to Pb Increases Metal Concentration in the Hippocampus

Analysis from microwave-induced plasma optical emission spectrometer (MIP OES) indicates higher Pb concentration in the hippocampus of animals that were exposed to metal (22.21 ± 0.77 mg·kg^−1^), when compared to the control group (9.71 ± 0.98 mg·kg^−1^). Due to the reduced amount of tissue, a pool of samples was made according to each experimental group. In order to verify the accuracy of the analysis, known amounts of Pb were added to the digested sample and, subsequently, a certain amount of Pb per MIP OES. The concentrations of 3.5 mg/L, 4.5 mg/L and 5.5 mg/L were added to the samples, resulting in a recovery percentage of 102.8%, 97.0% and 95.0%.

### 2.4. Effects of Long-Term Exposure to Pb on Oxidative Balance in the Hippocampus

**Figure 3:**
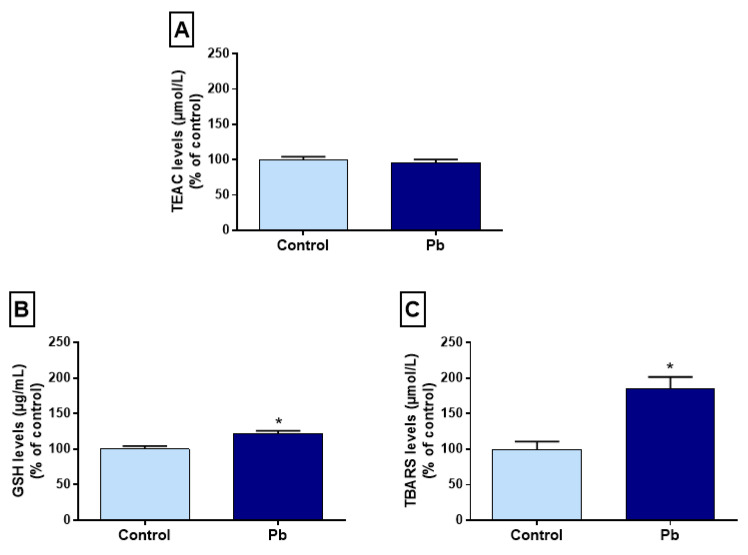
Effects of long-term exposure to lead acetate (Pb) on hippocampal oxidative biochemistry of rats exposed from adolescence to adulthood, expressed as percentage of control. (**A**) Trolox equivalent antioxidant capacity (TEAC); (**B**) glutathione (GSH); (**C**) thiobarbituric acid reactive substances (TBARS) levels. * *p* < 0.05 compared to the control group (Student’s *t*-test).

The evaluation of the Trolox equivalent antioxidant capacity (TEAC) did not show statistical difference among the groups (control: 100% ± 4.52%; Pb: 95.6% ± 4.94%; *p* = 0.5) (Figure 3A). It was observed that glutathione levels (GSH) in the hippocampus of exposed animals showed a significant increase in relation to the control group (control: 100% ± 4.17%; Pb: 185.2% ± 16.56%; *p* = 0.002) (Figure 3B). Animals exposed to Pb from adolescence to adulthood presented an increase in TBARS concentration in the hippocampus when compared to animals that received only H_2_O_dest_ (control: 100% ± 10.87%; Pb: 185.1% ± 16.56%; *p* = 0.001) (Figure 3C).

### 2.5. The Hippocampal Proteome was Significantly Changed after Pb Exposure

**Figure 4:**
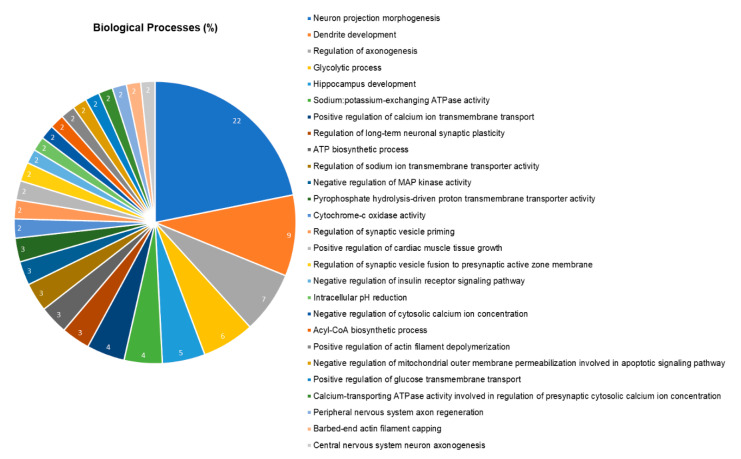
Functional distribution of proteins identified with differential expression in the hippocampus of rats exposed to lead acetate vs. control group. Protein categories based on Gene Ontology annotations on biologic processes. Significant terms and distribution according to the percentage of the number of genes. Protein access numbers were provided by UNIPROT. Genetic ontology was evaluated according to ClueGO plugin^®^ Cytoscape Software^®^ 3.6.

The hippocampal proteomic analysis identified a total of 252 proteins in different status of expression. Of these, 42 were identified only in the control group, while 51 were expressed only in the group exposed to Pb. The total of 159 proteins had altered expression, in which 108 were downregulated and 51 upregulated in relation to the Pb group. In addition, the protein were analyzed according to their biologic processes through gene ontology (Figure 4), being the five most affected processes related to neuron projection morphogenesis (22%), dendrite development (9%), regulation of axonogenesis (7%), glycolytic process (6%) and hippocampus development (5%). In Table 1 we highlighted the proteins used in the discussion and the complete result is available on supplementary material (Table S1).

### 2.6. Long-Term Exposure to Pb Promotes Neuronal Loss

**Figure 5:**
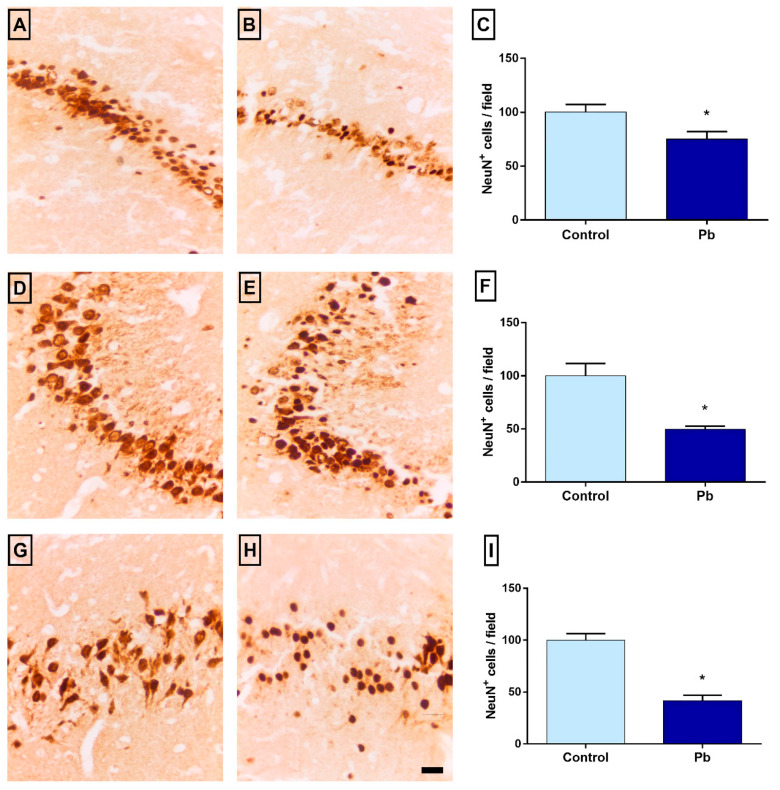
Immunostaining for anti-NeuN in the hippocampus. Representative photomicrographs of CA1, CA3 and hilus in control (**A**, **D** and **G**, respectively) and Pb-exposed group (**B**, **E** and **H**, respectively). Graphs with a quantitative representation of the differences between the number of NeuN^+^ cells in the respective areas: CA1 (**C**), CA3 (**F**) and hilus (**I**) expressed as mean ± Standard Error of Mean (S.E.M.) of the cell number per field in each region. * *p* < 0.05 compared to the control group (Student’s *t*-test). Scale bar: 20 μm.

The number of NeuN^+^ cells was decreased in Pb group when compared to control group in CA1 (control: 100 ± 7.31; Pb: 75 ± 6.87; *p* = 0.03), CA3 (control: 100 ± 11.66; Pb: 49 ± 2.98; *p* = 0.001) and hilus (control: 100 ± 6.25; Pb: 41.57 ± 5.35; *p* < 0.0001). Figure 5 shows the results of tissue evaluation in the hippocampus of animals exposed to Pb or H_2_O_dest_. Results are shown as a percentage of control.

## 3. Discussion

Our results show for the first time that long-term exposure to Pb, from adolescence to adulthood in rats, is able to promote increased levels of the metal in the hippocampus, oxidative stress and modulation of proteins related to cell protection, proteins from calcium–calmodulin signaling pathway and proteins related to synaptic transmission, associated with intense neuronal loss and damages to cognitive functions. To the best of our knowledge, this is the first time that it is showed in the literature that hippocampus, in this period of life, is also susceptible do Pb-induced impairments.

In this animal model, the Pb exposure occurred in a period between adolescence, marked in rats as the stage of sexual maturation, to adulthood, which begins around the 63^rd^ postnatal day [14]. This methodological choice is configured in a complementary approach to the various studies that focused mainly on investigating the effects of Pb exposure in earlier stages of life, since the intrauterine period, for example [12,15]. Investigating the neurotoxic effects of this metal at a later age favors an understanding focused on translational reality in occupational exposure. The Pb administration was performed through intragastric gavage, which allows greater control of the amount of metal administered according to the animal’s weight. The dose was selected based on previous studies, which identified that this protocol promoted a significant accumulation of Pb in the brain of rats [16,17], reinforced by previous experimentation in our group, showing that long-term exposure promotes Pb accumulation in the spinal cord and cerebellum of Wistar rats [18,19]. The absorption and distribution of Pb in this model were confirmed by measuring the metal in the nervous tissue, showing that the hippocampus of the exposed rats had significantly higher levels than the control group. Our results reinforce that not only the brain of a newborn or infant is susceptible to the harmful effects of Pb, but also when exposed between adolescence and adulthood.

To assess the possible changes triggered in the hippocampus by exposure to Pb, we chose to analyze parameters related to oxidative biochemistry. The pro-oxidant parameters approach is a consistent neurotoxicity assessment tool to investigate the deleterious effects of Pb exposure, exerted through oxidative stress, evidenced by induction of lipid peroxidation and generation of reactive oxygen species (ROS). In our study, although increased levels of MDA were found in the hippocampus of animals exposed to Pb, the total antioxidant capacity showed no difference between the groups. The overproduction of ROS induced by Pb may not be accompanied by an adequate response from the antioxidant system, however, it is considered an important triggering factor for cell death, such as apoptosis [20,21]. We believe that in the time and dose established in this study, the biochemical damage triggered was more influenced by the increase in the pro-oxidant damage, observed due to lipid peroxidation, than by a reduction in the hippocampal antioxidant defenses.

The change in biochemical homeostasis, with increased pro-oxidant factors, is also able to modulate the proteome, changing the redox control and the ability of proteasomes to remove irreversibly damaged proteins, in addition to preventing irreversible protein aggregation [22]. Among the groups of proteins with an important modulation function of the proteome, the heat shock proteins (HSP) have been widely associated with cell protection against stressor insults [23,24], being part of a large family of proteins that plays a pivotal role in cell survival, protein folding, cell signaling and regulating transcription factors [25,26]. The HSP90 family is the most abundant, presenting the HSP90α (P82995) as a faster cellular protection factor and HSP90β (P34058), responsible for long-term cellular adaptation [27]. Both members were found upregulated in our proteomic analysis, suggesting a mechanism of protection of hippocampal cells against long-term Pb exposure. On the other hand, we observed downregulation of HSP70 1A and 1B (P0DMW0 and P0DMW1, respectively) that are associated with the cytoskeleton protection and neuroprotection against incorrectly folded proteins in Alzheimer’s and Parkinson’s diseases [28,29]. Moreover, HSP are also involved in apoptosis prevention and oxidative stress responsiveness signaling [27].

In this perspective, our redox balance investigation showed an increase in lipid peroxidation levels, which could be associated with the modulation of cytochrome C (CyC) oxidase subunits (P00406 and P10888, both downregulated; P32551 and P10818, both upregulated; P12075, uniquely expressed in control) [30]. However, GSH levels were interestingly elevated in the Pb-exposed group, corroborating with our proteomic approach that showed Glutathione S Transferase P (P04906) exclusive expression in the exposed group. Then, we hypothesized that the LPO increase in this experimental model could be associated with other antioxidant systems failure, as the downregulation of carbonic anhydrase 2 (P27139), as well as mitochondrial impairments, once ATP synthase proteins were modulated (P10719, downregulated; P21571 and Q7TNJ2, exclusively expressed in Pb group).

The increase of reactive oxygen species (ROS) may drive to cell death, by oxidizing cell and organelles membranes [31]. In this way, the long-term exposure to Pb decreased the neuronal density in the hippocampus of adult rats as observed by anti-NeuN immunolabeling. Underlying this, our proteomic approach gave us suggestive evidences that the calcium–calmodulin (CC) pathway plays an important role in the apoptosis of neurons by upregulation of CC kinase II subunits (P11275, P08413, P15791 and P11730), besides the CyC modulation mentioned above. This issue had already been reported in the literature and associated with Pb-induced neurotoxicity, however, it is the first time in our knowledge that a whole proteomic profile of the hippocampus from animals exposed to Pb, indicated all pathways leading to a hippocampal dysfunction.

Underlying the memory and learning impairments caused by Pb-induced neurotoxicity, the proteomic investigation showed exclusive regulation of Excitatory amino acid transporter 1 (P24942) in control group and downregulation of proteins related to synapse transmission, as synapsin-1 (P09951) and 2 (Q63537), besides the synaptotagmin-1 (P21707), 2 (P29101) and 5 (P47861). Furthermore, synaptophysin (P07825) mediates the exocytosis–endocytosis of synaptic vesicles and regulates SNAREs complex formation, which is also composed of syntaxin proteins, as syntaxin-binding protein 1 (P61765) [32,33]. Both proteins were found upregulated in the hippocampus and, this fact can be related to an increase in neurotransmitter release in the synaptic cleft, suggesting a possible mechanism of excitotoxicity [34,35].

In addition, some evidences have suggested that the upregulation and expression of myelin protein constituents are associated with the demyelination process and with disturbance on cholesterol accumulation [36,37]. In addition, this cholesterol accumulation is may be associated with the exclusive expression of Apolipoprotein E (P02650) found in the control group. One of the roles played by P02650 is the reuse of membrane lipids during remyelination and neuronal repair [38,39].

The macrophage migration inhibitory factor (P30904) is a cytokine of 12.5 kD that regulates the immune response and has the expression increased in traumatic brain injuries and other pathological conditions as multiple sclerosis, meningitis and Alzheimer’s disease [40,41,42,43] and it was found upregulated in our proteomic approach. It is important to highlight that cerebral injuries triggered by mechanical, immunological, diseases or even by metals insults, can also display astrocytes reactivity, that is accompanied by glial fibrillary acidic protein (GFAP, P47819) upregulation [44,45], which was observed in the exposed group. In addition, the protein S100B (P04631), found downregulated in the hippocampus of rats exposed to Pb, exerts important roles in cell metabolism and cell proliferation and differentiation [46] and its downregulation has been associated with persistently infected brains, followed by astrocytosis [47], i.e., constant glial responsiveness.

Our investigations also aimed to elucidate whether the oxidative and proteomic biochemical changes would be capable of culminating in hippocampal neurodegeneration of mature neurons. In our results, the reduction in neuronal density found in the three hippocampal regions evaluated led us to the understanding that this pattern of neurodegeneration is associated with functional impairments in exposed animals. The areas evaluated in this work are part of the neuronal circuitry responsible for several mnemonic functions investigated, CA1, e.g., from its projections into the subiculum, connects with the neocortex [48] and associate the hippocampus with the stabilization function of the cortical representation of events learned from a late consolidation [49].

Data from functional investigations resulting from Pb exposure include memory changes and although it is not possible to approach hippocampal function in isolation, it is possible to select behavioral tests that are reliable in identifying the consequences of long-term exposure to Pb. The object recognition test was selected as an important tool to assess possible changes resulting from hippocampal injuries. One aspect of this test, the judgment of the previous occurrence of individual items depends on the perirhinal cortex, but the associations of context and temporal aspects depend on the anatophysiological interactions among the hippocampus and the perirhinal and prefrontal cortices, highlighting the participation of the hippocampus in processes involving information with spatial components [50] or when the memory contains a time component [51].

In addition to the subject’s cognitive skills rescue regarding the tasks that assess the exploration of environments or, in this case, objects [52], one of the main advantages of the object recognition test is that it is based on the natural tendency of rodents to explore novelties, not requiring many training sessions. In our research, we started with the protocol that provides a short-term memory assessment, which has changed in animals exposed to Pb acetate. Performing this assay, we observed that mice exposed to Pb present a shorter investigation and recognition time, indicating a change in short-term memory, implicated with hippocampal functions, the target of our study. Memory consolidation is a process for stabilizing short-term memory and forming long-term memory. The mechanisms related to the consolidation of object recognition memory involve several brain areas, including the hippocampus, as identified from an investigation of gene expression, which points out that the formation of this type of memory induces the expression of the *c-fos* gene in the hippocampus, especially in CA1 and CA3, in addition to the medial, perirhinal and insular prefrontal cortices [53,54,55].

The inhibitory avoidance test indicates that long-term exposure to Pb causes impaired memory retention compared to the control group. There is evidence that animals exposed to metal need more stimulation on their feet to reach the learning criteria [56], although our results for the memory retention tests, performed 24 h later, do not differ between the groups evaluated. Such difference in results can be justified in the difference between the doses used and the time of administration of the metal, different in the two proposals.

## 4. Materials and Methods

### 4.1. Experimental Animals

**Figure 6:**
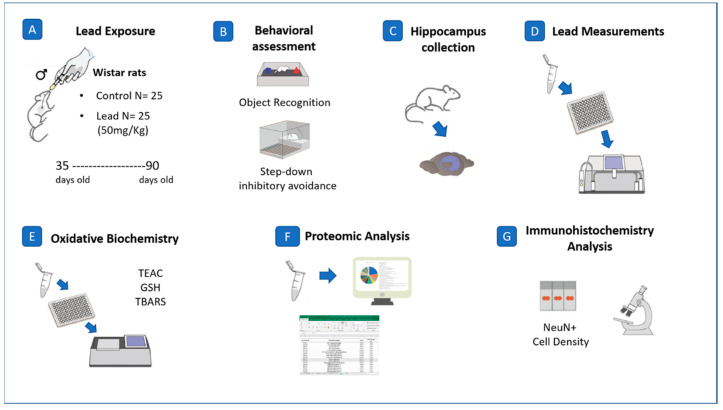
Methodological steps of this research. (**A**) description of the Pb exposure model and sample size; (**B**) behavioral tests elected for this investigation: object recognition test and step-down inhibitory avoidance test; (**C**) samples collection; (**D**) measurement of Pb levels; (**E**) analyses of oxidative biochemistry, through pro and antioxidant parameters; (**F**) proteomic profile performed by mass spectrometry; (**G**) immunohistochemical analysis for NeuN^+^ cells density.

Male Wistar rats (*n* = 50; Control = 25; Pb = 25) with 35 days of life at the beginning of the experiments were used in this study. All animals were euthanized on the 91^st^ postnatal day. They were housed in plastic collective cages, fed with balanced feed and water ad libitum, at a temperature of 25 °C, dark/light cycle of 12 h (06:00–18:00). This project was submitted to the ethics committee on the use of animals of the Federal University of Pará (CEUA—UFPA), approved under CEUA number 2237110716. All the procedures adopted in this research are summarized schematically in a methodological figure (Figure 6).

### 4.2. Lead Exposure and Body Mass Registration

The experimental animals were exposed to Pb acetate at 50 mg/kg (Sigma-Aldrich, St. Louis, MO, USA) [17] from the 35th day to the 90th postnatal day by intragastric gavage. The control group received distilled water (H_2_O_dest._), in the same volume as the exposed group. Gavage administration was used by our group in different models of exposure to toxicants [57,58,59]. The animals were weekly weighed for dose adjustment and verification of possible changes in body mass due to metal effects.

### 4.3. Behavioral Assays

In order to evaluate the mnemonic patterns presented after exposure to Pb, the animals of both groups were subjected to behavioral tests developed and performed to identify possible alterations in memory function. All animals were submitted to behavioral tests.

#### 4.3.1. Object Recognition

The object recognition consists of a black wooden arena (100 × 100 × 30 cm), virtually divided into twenty-five quadrants with equal dimensions (20 × 20 cm). The protocol was adapted [60] and consists in the habituation phase, the animal was placed in the empty apparatus for freely exploratory movement for 5 min. After 30 min, animals were positioned in the apparatus, containing two identical cube-shaped objects (C1 and C2), located in opposites corners (10 cm away from the walls). At this stage, the animals were able to investigate spontaneously the objects for 3 min. In the test phase (30 min after a training session), an identical copy of the familiar object (C3) and a new object (X) were placed in the arena at the same locations previously occupied by C1 and C2 objects in the training phase. Each animal was placed on the center of the apparatus and freely exploratory behavior was allowed for 3 min.

The investigation time of the objects by animals was registered in the training and test session. The investigation was defined as the moment when the head of the animal faced the object at a distance equal to or less than 4 cm (adapted [61]). The analyses were performed considering the total exploration time spent on the two objects in the training phase (C1 + C2). The recognition index was defined by the difference in the time of exploration between the new object (X) and the familiar member (C3) divided by the total time spent in the exploration between the same objects in the test phases (X − C3) / (X + C3). The apparatus was cleaned with alcohol 10%. The data evaluated through the software ANY-maze TM (Stoelting Co., San Diego, CA, USA).

#### 4.3.2. Step-Down Inhibitory Avoidance Test

For this test, the animals were inserted into a device consisting of an acrylic box (50 × 25 × 25 cm) containing a floor composed of parallel metal bars (1 mm in diameter), with spaces of 1 cm between them, connected to an electric stimulator. It also has a platform not connected to the electric stimulator (7 × 2.5 cm). In the habituation session, the animals were placed on the first day next to the wall not connected to electricity and the step-down latency, i.e., the time to descent from the platform to the grid (with the four legs) was recorded [62]. Thirty minutes later, the animals were once again placed on the platform next to the wall not connected to electricity and they were stimulated with a slight shock to the four paws (0.4 mA) for 1 s. On the second day, the same protocol was performed, however, without electric stimulus for long-term memory evaluation, performing the same procedure, recording latency for platform descent. We consider as a good performance in this test if the animals kept the ability of remembering that the platform would be a safe place, instead of descending to the electrified grid [63].

### 4.4. Quantification of Pb in Neural Parenchyma

Ten animals per group were anesthetized with ketamine hydrochloride (90 mg/kg) and xylazine hydrochloride (9 mg/Kg), the brains removed from the cranial box and the structures rapidly dissected in cold places and frozen in liquid nitrogen, kept in ultra-freezer (−80 °C) until the moment of analysis. Subsequently, the tissues were lyophilized. After lyophilization, as the amount of mass obtained was small, a pool of samples per group was performed. A mass of each sample was weighed and transferred to the digestion bottle, adding 4.0 mL HNO_3_ (14 mol/L), 2.0 mL H_2_O_2_ (35% *w/w*), and 2.0 mL ultrapure H_2_O. The digestion of the samples was performed in triplicate, in a microwave oven with a cavity (START E, Milestone, Sorisole, Italy) at a temperature of 180 °C and power of 800 W, for 25 min.

After digestion, the solutions were transferred to volumetric vials, maintaining a final volume of 40 μL with ultrapure water. For Pb determination, the samples were diluted to the final acidity of 5.0%.

### 4.5. Oxidative Biochemistry Analyses

To evaluate the effect of Pb exposure on the balance between endogenous antioxidant capacity and the production of reactive oxygen species, several biochemical assays were performed in the hippocampus.

#### 4.5.1. Obtaining and Preparing Samples

One hemisphere of each hippocampus from the animals described above was dissected and conducted to oxidative biochemistry analyses. Each sample was weighed and placed in microtubes with PBS at a ratio of 1:10 and subsequently sonicated. After sonication, the samples were centrifuged, the supernatant collected and stored at −80 °C until the moment of analysis.

#### 4.5.2. Determination of Trolox Equivalent Antioxidant Capacity (TEAC)

It is based on antioxidant inhibition of radical cation ABTS+•. The addition of antioxidants to this preformed cation radical reduces it again to ABTS, on a scale dependent on antioxidant capacity, antioxidant concentration and duration of the reaction. The TEAC was determined according to the antioxidant capacity equivalent to acid (±) -6-hydroxy-2,5,7,8-tetramethylchroman-2-carboxylic (Trolox, Sigma-Aldrich), according to the methodology modified by [64]. Exactly 2970 μL of the ABTS+ working solution was placed in the bucket, followed by the first reading (T0). Then, 100 μL of sample were transferred to test tubes containing 900 μL of water. From the samples, aliquots of 30 μL were removed, transferred to the bucket containing the radical, and after 5 min, the second reading was performed. The reaction was measured by spectrophotometry by observing the change in absorbance read at 734 nm for five minutes. Thus, the total antioxidant activity of the samples was determined, and its relationship with Trolox reactivity was calculated as standard, by performing a standard curve under the same conditions. Antioxidant capacity was expressed in μmol/L.

#### 4.5.3. Determination of Glutathione

Glutathione (GSH) was determined according to the methodology adapted from [65]. The assay is based on the reaction of GSH with a chromogen, acid-5,5-dithiobis-2-nitrobenzoic (DTNB or Ellman reagent; Sigma-Aldrich), generating yellow-colored 2-nitro-5-mercapto-benzoic acid (TNB), which is quantified by 412 nm wavelength spectrophotometry. Aliquots of 100 μL were removed from the samplesand transferred to a test tube containing 900 μL of water. Then, 20 μL of the samples were transferred to a test tube containing 20 μL of distilled water and 4 mL of buffer solution. From these tubes, 3 mL of samples were removed and transferred to the bucket, followed by the 1st reading of the sample (T0) at 412 nm. Soon after, 100 μL of DTNB solution (5.5-dithio-bis(2-nitrobenzoic acid)) was added to the sample and after 3 min, the second reading (T3) was performed to determine the GSH concentration expressed in μg/mL.

#### 4.5.4. Thiobarbituric Acid Reactive Substances (TBARS)

It allows the measurement of lipid peroxidation and was used as an indicator of oxidative stress. In this method, malondialdehyde (MDA) formed during lipid peroxidation and other substances react with thiobarbituric acid (TBA) at low pH and high temperature, forming the pink MDA–TBA complex, detected by spectrophotometry at 535 nm. The technical procedure was performed according to the methods adapted by [66]. An aliquot of 100 μL from the samples and transferred to test tubes containing 900 μL of water. Then, 0.5 mL of the samples were transferred to test tubes containing 1 mL of TBA solution (10 nM). Then, these tubes were placed in a water bath at 94 °C for 60 min. After this period, 4 mL of n-butyl alcohol (Sigma-Aldrich) was added to the tubes, stirring in vortex, followed by centrifugation at 175× *g* (15 min). Subsequently, 3 mL of supernatant were transferred to the bucket, and then the spectrophotometric reading was performed at 535 nm. The concentration of TBARS was expressed in μmol/L.

At the end of the biochemical assays, the results of TEAC, GSH and TBARS were transformed and expressed in percent of the control groups.

### 4.6. Proteomic Approach

For proteomic analyses (six animals per group), the samples were collected and immediately frozen in liquid nitrogen and stored at −80 °C until the next steps. First, we performed the protein extraction, followed by digestion, purification/desalinization and then mass spectrometry analysis.

#### 4.6.1. Protein Extraction, Digestion and Purification

The current protocol was previously published by our group in [44,67,68]. First, the hippocampus from two animals were pooled and each group was carried out in biologic triplicate. Then, the pooled samples were cryofractured in liquid nitrogen by a cryogenic mill, followed by protein extraction with lysis buffer [7-M urea, 2-M thiourea and 40-mM dithiothreitol (DTT)] diluted in ammonium bicarbonate (AmBic, 50 mM) solution, under constant shaking at 4 °C. After that, the samples were centrifuged at 14,000 rpm for 30 min at 4 °C, the supernatant was collected to quantify the amount of proteins by Bradford’s method and proceed the following steps. A total of 50 μg of protein was collected and the correspondent volume was filled with AmBic to reach the final volume of 50 μL (1 μg/μL). In each sample were added 10 μL of 50-mM AmBic and 25 μL of 0.2% RapiGEST™ (Waters Co., Manchester, UK) and incubated for 30 min at 37 °C. Then, 2.5 μL of 100-mM DTT was added and incubated at 37 °C for 40 min, followed by incubation with 2.5 μL of 300-mM iodoacetamide for 30 min at room temperature. To proceed the protein digestion, we added 10 μL of trypsin for 14 h at 37 °C with subsequent addition of 10 µL of 5% trifluoroacetic acid and incubation for 90 min at 37 °C. The samples were centrifuged at 14,000 rpm for 30 min at 6 °C, the supernatants were collected and purified using C18 spin columns (Pierce™). After purification, the samples were concentrated to an approximate concentration of 1 μg/μL and then, they were resuspended in 12 μL of ADH (1 pmol/μL) + 108 μL of 3% acetonitrile and 0.1% formic acid for mass spectrometry analyses.

#### 4.6.2. Mass Spectrometry and Bioinformatic Analyses

The mass spectrometry system used for the proteomic approach was a nanoAcquity UPLC-Xevo QTof MS system (Waters, Manchester, UK), using the ProteinLynx Global SERVER (PLGS) software (Waters, Milford, MA, USA), as previously described by [69] after downloading Uniprot database. The PLGS software applied the Monte-Carlo algorithm to obtain the difference of protein expression between the groups, considering *p* < 0.05 for downregulated proteins and 1 − *p* > 0.95 for upregulated proteins. After the identification and categorization of proteins, the Cytoscape 3.6.1 (Java^®^) software (National Institute of General Medical Sciences, Rockville, MD, USA) was used for bioinformatics analyses with the ClueGO plugin for the determination of biologic processes groups [70].

### 4.7. Perfusion, Histological and Immunohistochemistry Procedures

To investigate the possible neuronal death triggered in this experimental model, 24 h of behavioral assays, 9 animals from each group were randomly selected and submitted to perfusion, histological processing and immunohistochemical evaluation for immunostaining of mature neurons in the hippocampus. For this investigation, we selected the anti-NeuN marking, previously described in our studies [18,71]. For this, they were anesthetized with ketamine hydrochloride (90 mg/kg) and xylazine hydrochloride (9 mg/kg) and perfused through the left ventricle of the heart with 0.9% heparinized saline solution, followed by 4% paraformaldehyde. The brains were removed, postfixed for 4 h in Bouin’s solution and subsequently processed in alcohol and xylol battery and embedded in Paraplast (McCormick Scientific, Baltimore, MD, USA). Coronal sections of 7-μm-thickness of the dorsal hippocampus were obtained through the use of microtome and intended for immunohistochemical analysis. Soon after the microtomy, all sections were mounted directly on slides prepared with poly D-lysine. To increase the adhesion of the sections, the slides were kept in an oven at a temperature of 55 °C for at least 24 h before any other histological procedure. For immunohistochemistry, the slides were submitted to deparaffinization in xylol solutions and hydrated in alcoholic solutions in decreasing concentrations (ABS II, ABS I, 90, 80 and 70%). They were then washed in distilled water and PBS, also through endogenous peroxidase blockade in 3% hydrogen peroxide solution in methanol. For antigenic recovery, the samples were preheated in citrate buffer with pH 6.0, previously heated to 60 °C for 20 min. After that, sections were further allowed to cool for 20 min and incubated in 1% hydrogen peroxide solution (H_2_O_2_) in methanol for 20 min to inhibition of endogenous peroxidase activity. Then, sections were rinsed three times in 0.1-M PBS/Tween (Sigma-Aldrich^®^, St. Luis, MO, USA) solution for 5 min and incubated with 10% normal horse serum and 3% bovine serum albumin (BSA) in PBS for two hours. Without further rinsing, sections were incubated overnight with the primary antibody anti-NeuN in PBS (1:100, Chemicon, Millipore, Bedford, MA, USA), rinsed in PBS/Tween solution for five minutes (three times) and incubated with biotinylated horse anti-mouse secondary antibody (Vector Laboratories, Burlingame, CA, USA). Sections were rinsed again for five minutes (three times) and incubated in the avidin–biotin–peroxidase complex (ABC Kit, Vector Laboratories) for two hours. Sections were rinsed four times and revealed with 3.3’ diaminobenzidine (DAB), rinsed two times in 0.1-M PBS, dehydrated using alcohols and xylene and coverslipped with Entellan (Merck^®^, Darmstadt, Germany).

For the quantitative evaluation of NeuN^+^ cell density, immunohistochemical marking counts were performed using a Nikon Eclipse E200 binocular microscope (Nikon, Minato, Japan), using a 0.00625 mm² count grid coupled to one of the oculars, at a 40× lens. Three sections per animal were counted in areas CA1, CA3 and hilus. All sections had a minimum distance between them, with an attempt to obtain a more complete and representative analysis of the rostrocaudal extension of the region.

## 5. Conclusions

Impaired hippocampal structure and function of rats exposed from adolescence to adulthood to Pb acetate reinforces the vulnerability of the hippocampus to this metal, showing changes in the expression of important proteins to the physiological functions of nervous tissue, an increase in the level of lipid peroxidation, altering the redox balance of the hippocampus, in addition to a significant neuronal loss, which in the combination of these factors, culminated in a functional damages in the mnemonic behavior.

## Figures and Tables

**Table 1:** Identified proteins with expression significantly altered in the hippocampus of rats of exposed group (Pb) vs. Control.

Accession Id ^a^	Protein Description	Score	Fold Change
			Pb
P0DMW1	Heat shock 70 kDa protein 1B	4870	−0.932
P09951	Synapsin-1	18,756	−0.914
P0DMW0	Heat shock 70 kDa protein 1A	4870	−0.896
P10719	ATP synthase subunit beta, mitochondrial	232,442	−0.835
P04631	Protein S100-B	205,290	−0.835
Q63537	Synapsin-2	41,092	−0.819
P47861	Synaptotagmin-5	2245	−0.795
P29101	Synaptotagmin-2	2245	−0.763
P21707	Synaptotagmin-1	20,604	−0.748
P10888	Cytochrome c oxidase subunit 4 isoform 1, mitochondrial	14,314	−0.625
P00406	Cytochrome c oxidase subunit 2	42,862	−0.538
P47819	Glial fibrillary acidic protein	14,060	1.062
P61765	Syntaxin-binding protein 1	59,845	1.073
P30904	Macrophage migration inhibitory factor	38,694	1.094
P11275	Calcium/calmodulin-dependent protein kinase type II subunit alpha	181,954	1.105
P15791	Calcium/calmodulin-dependent protein kinase type II subunit delta	62,682	1.105
P08413	Calcium/calmodulin-dependent protein kinase type II subunit beta	80,840	1.127
P11730	Calcium/calmodulin-dependent protein kinase type II subunit gamma	66,045	1.127
P34058	Heat shock protein HSP 90-beta	16,345	1.197
P10818	Cytochrome c oxidase subunit 6A1, mitochondrial	30,209	1.284
P07825	Synaptophysin	42,961	1.310
P32551	Cytochrome b-c1 complex subunit 2, mitochondrial	4014	1.323
P02650	Apolipoprotein E	4894	−
P21571	ATP synthase-coupling factor 6, mitochondrial	31,889	+
Q7TNJ2	ATP-binding cassette sub-family A member 7	3891	+
P27139	Carbonic anhydrase 2	12,017	−
P12075	Cytochrome c oxidase subunit 5B, mitochondrial	23,587	−
P24942	Excitatory amino acid transporter 1	27,055	−
P04906	Glutathione S-transferase P	9312	+
**+ 219 proteins with different status of regulation**			

^a^ Accession ID according to the Uniport.org database. Positive and negative values of fold change indicate up- and downregulated proteins, respectively. Signs of + or − indicates exclusive expression in the Pb group and the control group, respectively. Results of the comparison between the Pb group and the control group.

## Supplementary Materials

Supplementary materials can be found at https://www.mdpi.com/1422-0067/21/18/6937/s1. Table S1. Identified proteins with expression significantly altered in the hippocampus of rats of exposed group (Pb) vs. Control.

## Abbreviations

CC	Calcium-calmodulin
CyC	Cytochrome C
GSH	Glutathione
HSP	Heat shock proteins
MDA	Malondialdehyde
MIP OES	Microwave-induced plasma-optical emission spectrometer
Pb	Lead
PbS	Galena
ROS	Reactive oxygen species
TBARS	Thiobarbituric acid reactive substances
TEAC	Trolox equivalent antioxidant capacity
