# Supplementary material for: Hippocampal Impairment Triggered by Long-Term Lead Exposure from Adolescence to Adulthood in Rats: Insights from Molecular to Functional Levels
Source: Int J Mol Sci. 2020 Sep 21;21(18):6937. doi: 10.3390/ijms21186937 (PMC7554827; doi:10.3390/ijms21186937)
Supplement: Supplementary file 1 [file ijms-21-06937-s001.pdf]

International Journal of Molecular Sciences

MDPI

Table 1. Identified proteins with expression significantly altered in the hippocampus of rats of exposed group (Pb) *vs*. Control.

Accession Id [a]	Protein Description	Score	Fold Change Pb
P35213	**14-3-3 protein beta/alpha**	85420	−0.835
P62260	**14-3-3 protein epsilon**	96570	−0.878
P68511	**14-3-3 protein eta**	85420	−0.844
P68255	**14-3-3 protein theta**	85420	−0.835
P63102	**14-3-3 protein zeta/delta**	105051	−0.803
P13233	**2',3'-cyclic-nucleotide 3'-phosphodiesterase**	151400	1.405
P68035	**Actin, alpha cardiac muscle 1**	442584	−0.942
P68136	**Actin, alpha skeletal muscle**	441060	−0.970
P62738	**Actin, aortic smooth muscle**	438270	−0.970
P60711	**Actin, cytoplasmic 1**	630104	−0.942
P63259	**Actin, cytoplasmic 2**	630104	−0.942
P63269	**Actin, gamma-enteric smooth muscle**	438270	−0.951
Q05962	**ADP/ATP translocase 1**	60100	−0.554
Q09073	**ADP/ATP translocase 2**	49102	−0.482
P84079	**ADP-ribosylation factor 1**	34675	−0.644
P84082	**ADP-ribosylation factor 2**	22412	−0.644
P61206	**ADP-ribosylation factor 3**	34675	−0.619
P61751	**ADP-ribosylation factor 4**	22412	−0.670
P84083	**ADP-ribosylation factor 5**	22412	−0.625
P04764	**Alpha-enolase**	46219	−0.951
P23565	**Alpha-internexin**	9478	1.062
P37377	**Alpha-synuclein**	89619	−0.771
P13221	**Aspartate aminotransferase, cytoplasmic**	23661	1.083
P00507	**Aspartate aminotransferase, mitochondrial**	46049	1.116
P10719	**ATP synthase subunit beta, mitochondrial**	232442	−0.835
P85969	**Beta-soluble NSF attachment protein**	9638	1.419
Q63754	**Beta-synuclein**	66842	−0.779
P11275	**Calcium/calmodulin-dependent protein kinase type II subunit alpha**	181954	1.105
P08413	**Calcium/calmodulin-dependent protein kinase type II subunit beta**	80840	1.127
P15791	**Calcium/calmodulin-dependent protein kinase type II subunit delta**	62682	1.105

P11730	**Calcium/calmodulin-dependent protein kinase type II subunit gamma**	66045	1.127
P0DP29	**Calmodulin−1**	96662	−0.923
P0DP30	**Calmodulin-2**	96662	−0.932
P0DP31	**Calmodulin-3**	96662	−0.905
Q5U206	**Calmodulin-like protein 3**	22038	−0.869
P11442	**Clathrin heavy chain 1**	19100	1.162
P45592	**Cofilin−1**	95634	−0.942
Q63198	**Contactin−1**	5081	1.310
P07335	**Creatine kinase B-type**	133047	−0.844
P25809	**Creatine kinase U-type, mitochondrial**	9299	1.127
P32551	**Cytochrome b-c1 complex subunit 2, mitochondrial**	4014	1.323
P00406	**Cytochrome c oxidase subunit 2**	42862	−0.538
P10888	**Cytochrome c oxidase subunit 4 isoform 1, mitochondrial**	14314	−0.625
P10818	**Cytochrome c oxidase subunit 6A1, mitochondrial**	30209	1.284
Q6P6R2	**Dihydrolipoyl dehydrogenase, mitochondrial**	6786	−0.583
P21575	**Dynamin−1**	16920	−0.951
P39052	**Dynamin-2**	7306	1.127
Q08877	**Dynamin-3**	5050	1.297
P06761	**Endoplasmic reticulum chaperone BiP**	17487	−0.878
P31596	**Excitatory amino acid transporter 2**	135403	−0.432
P09117	**Fructose-bisphosphate aldolase C**	78961	−0.869
P47819	**Glial fibrillary acidic protein**	14060	1.062
Q6P6V0	**Glucose-6-phosphate isomerase**	5393	1.162
P10860	**Glutamate dehydrogenase 1, mitochondrial**	19715	1.162
P04797	**Glyceraldehyde-3-phosphate dehydrogenase**	388167	−0.887
Q9ESV6	**Glyceraldehyde-3-phosphate dehydrogenase, testis-specific**	43473	−0.387
Q63942	**GTP-binding protein Rab-3D**	23651	−0.712
P08753	**Guanine nucleotide-binding protein G(i) subunit alpha**	77246	−0.852
P10824	**Guanine nucleotide-binding protein G(i) subunit alpha−1**	79753	−0.811
P04897	**Guanine nucleotide-binding protein G(i) subunit alpha-2**	77246	−0.852
P54311	**Guanine nucleotide-binding protein G(I)/G(S)/G(T) subunit beta−1**	16927	1.051
P54313	**Guanine nucleotide-binding protein G(I)/G(S)/G(T) subunit beta-2**	18410	1.150
P59215	**Guanine nucleotide-binding protein G(o) subunit alpha**	151399	1.433
P38406	**Guanine nucleotide-binding protein G(olf) subunit alpha**	77246	−0.844
P63095	**Guanine nucleotide-binding protein G(s) subunit alpha isoforms short**	77246	−0.844
Q63803	**Guanine nucleotide-binding protein G(s) subunit alpha isoforms XLas**	77246	−0.852

P29348	**Guanine nucleotide-binding protein G(t) subunit alpha-3**	77246	−0.852
Q63210	**Guanine nucleotide-binding protein subunit alpha−12**	77156	−0.844
Q6Q7Y5	**Guanine nucleotide-binding protein subunit alpha−13**	77156	−0.861
P0DMW0	**Heat shock 70 kDa protein 1A**	4870	−0.896
P0DMW1	**Heat shock 70 kDa protein 1B**	4870	−0.932
Q5XHZ0	**Heat shock protein 75 kDa, mitochondrial**	15232	1.246
P82995	**Heat shock protein HSP 90-alpha**	13791	1.174
P34058	**Heat shock protein HSP 90-beta**	16345	1.197
P01946	**Hemoglobin subunit alpha−1/2**	602284	−0.763
P02091	**Hemoglobin subunit beta−1**	219977	−0.657
P11517	**Hemoglobin subunit beta-2**	190718	−0.619
P05708	**Hexokinase−1**	3920	1.221
Q00715	**Histone H2B type 1**	72080	1.271
P62804	**Histone H4**	86771	1.150
P04642	**L-lactate dehydrogenase A chain**	53083	−0.961
P30904	**Macrophage migration inhibitory factor**	38694	1.094
O88989	**Malate dehydrogenase, cytoplasmic**	58867	−0.923
P04636	**Malate dehydrogenase, mitochondrial**	106122	−0.869
Q63560	**Microtubule-associated protein 6**	2813	1.377
P02688	**Myelin basic protein**	534696	1.041
P60203	**Myelin proteolipid protein**	332656	2.586
Q63345	**Myelin-oligodendrocyte glycoprotein**	63588	−0.748
P13596	**Neural cell adhesion molecule 1**	2018	−0.795
O35095	**Neurochondrin**	16987	−0.779
P19527	**Neurofilament light polypeptide**	9132	−0.923
Q05982	**Nucleoside diphosphate kinase A**	61455	1.062
P10111	**Peptidyl-prolyl cis-trans isomerase A**	72155	1.083
O35244	**Peroxiredoxin-6**	20269	1.246
P31044	**Phosphatidylethanolamine-binding protein 1**	53659	−0.887
P16617	**Phosphoglycerate kinase 1**	30992	1.073
P25113	**Phosphoglycerate mutase 1**	49565	1.030
P09626	**Potassium-transporting ATPase alpha chain 1**	22935	−0.619
P54708	**Potassium-transporting ATPase alpha chain 2**	76040	−0.264
P62963	**Profilin−1**	57416	1.185
P63319	**Protein kinase C gamma type**	5667	1.822
P04631	**Protein S100-B**	205290	−0.835

P49432	**Pyruvate dehydrogenase E1 component subunit beta, mitochondrial**	20003	1.139
P11980	**Pyruvate kinase PKM**	130661	−0.861
P50399	**Rab GDP dissociation inhibitor beta**	46816	−0.914
P35281	**Ras-related protein Rab−10**	12255	−0.684
P35284	**Ras-related protein Rab−12**	10865	−0.670
P35286	**Ras-related protein Rab−13**	3247	−0.651
P61107	**Ras-related protein Rab−14**	10865	−0.664
P35289	**Ras-related protein Rab−15**	10847	−0.677
Q6NYB7	**Ras-related protein Rab−1A**	12255	−0.684
P10536	**Ras-related protein Rab−1B**	12255	−0.691
P51156	**Ras-related protein Rab-26**	10865	−0.670
Q5U316	**Ras-related protein Rab-35**	12517	−0.684
P63012	**Ras-related protein Rab-3A**	33157	−0.705
Q63941	**Ras-related protein Rab-3B**	21898	−0.705
P62824	**Ras-related protein Rab-3C**	21898	−0.712
Q53B90	**Ras-related protein Rab-43**	10865	−0.670
P05714	**Ras-related protein Rab-4A**	10865	−0.670
P51146	**Ras-related protein Rab-4B**	10865	−0.664
Q9WVB1	**Ras-related protein Rab-6A**	9041	−0.670
P35280	**Ras-related protein Rab-8A**	12255	−0.677
P70550	**Ras-related protein Rab-8B**	12255	−0.684
Q5XI73	**Rho GDP-dissociation inhibitor 1**	83284	1.139
P63329	**Serine/threonine-protein phosphatase 2B catalytic subunit alpha isoform**	21080	1.105
P02770	**Serum albumin**	3164	−0.869
P06685	**Sodium/potassium-transporting ATPase subunit alpha−1**	121181	−0.527
P06686	**Sodium/potassium-transporting ATPase subunit alpha-2**	153567	−0.517
P06687	**Sodium/potassium-transporting ATPase subunit alpha-3**	203866	−0.273
Q64541	**Sodium/potassium-transporting ATPase subunit alpha-4**	77334	−0.492
P07340	**Sodium/potassium-transporting ATPase subunit beta−1**	152988	−0.379
P09951	**Synapsin−1**	18756	−0.914
Q63537	**Synapsin-2**	41092	−0.819
P07825	**Synaptophysin**	42961	1.310
P60881	**Synaptosomal-associated protein 25**	16493	−0.779
P21707	**Synaptotagmin−1**	20604	−0.748
P29101	**Synaptotagmin-2**	2245	−0.763
P47861	**Synaptotagmin-5**	2245	−0.795

P61765	**Syntaxin-binding protein 1**	59845	1.073
P01830	**Thy−1 membrane glycoprotein**	12202	1.221
P48500	**Triosephosphate isomerase**	113496	−0.827
P68370	**Tubulin alpha−1A chain**	629522	−0.852
Q6P9V9	**Tubulin alpha−1B chain**	575866	−0.852
Q6AYZ1	**Tubulin alpha−1C chain**	469951	−0.844
Q68FR8	**Tubulin alpha-3 chain**	533024	−0.861
Q5XIF6	**Tubulin alpha-4A chain**	319537	−0.844
Q6AY56	**Tubulin alpha-8 chain**	264255	−0.861
P85108	**Tubulin beta-2A chain**	1124869	−0.852
Q3KRE8	**Tubulin beta-2B chain**	1123519	−0.861
Q4QRB4	**Tubulin beta-3 chain**	723020	−0.852
Q6P9T8	**Tubulin beta-4B chain**	1042088	−0.980
P69897	**Tubulin beta-5 chain**	1033610	1.030
Q00981	**Ubiquitin carboxyl-terminal hydrolase isozyme L1**	20178	1.105
P63045	**Vesicle-associated membrane protein 2**	26914	−0.811
Q9QUL6	**Vesicle-fusing ATPase**	14577	1.062
Q9R1Z0	**Voltage-dependent anion-selective channel protein 3**	26793	1.174
P62815	**V-type proton ATPase subunit B, brain isoform**	12704	1.209
Q6PCU2	**V-type proton ATPase subunit E 1**	12118	−0.896
Q5RKI0	**WD repeat-containing protein 1**	15422	1.246
P26772	**10 kDa heat shock protein, mitochondrial**	55215	+
Q62878	**3 beta-hydroxysteroid dehydrogenase/Delta 5-->4-isomerase type 4**	5267	−
P07953	**6-phosphofructo-2-kinase/fructose-2,6-bisphosphatase 1**	4794	−
P11030	**Acyl-CoA-binding protein**	100395	−
P07943	**Aldo-keto reductase family 1 member B1**	7599	−
Q64563	**All-trans-retinol dehydrogenase [NAD(+)] ADH4**	24811	−
Q63028	**Alpha-adducin**	6503	+
P54921	**Alpha-soluble NSF attachment protein**	17613	+
Q02356	**AMP deaminase 2**	4914	+
O08838	**Amphiphysin**	4776	−
P18484	**AP-2 complex subunit alpha-2**	6539	+
P62944	**AP-2 complex subunit beta**	4673	+
P84092	**AP-2 complex subunit mu**	4908	−
P02650	**Apolipoprotein E**	4894	−
P21571	**ATP synthase-coupling factor 6, mitochondrial**	31889	+

Q7TNJ2	**ATP-binding cassette sub-family A member 7**	3891	+
Q3BCU4	**Blood vessel epicardial substance**	5339	−
P55068	**Brevican core protein**	10527	+
D3Z8E6	**Calmodulin-regulated spectrin-associated protein 1**	3976	−
P27139	**Carbonic anhydrase 2**	12017	−
Q9EQV9	**Carboxypeptidase B2**	4326	−
Q5M7A7	**CB1 cannabinoid receptor-interacting protein 1**	33730	+
P86182	**Coiled-coil domain-containing protein 22**	5304	+
P20788	**Cytochrome b-c1 complex subunit Rieske, mitochondrial**	28187	+
P12075	**Cytochrome c oxidase subunit 5B, mitochondrial**	23587	−
Q9JHU0	**Dihydropyrimidinase-related protein 5**	11356	−
O55096	**Dipeptidyl peptidase 3**	4656	+
O88797	**Disabled homolog 2**	4094	+
Q8VHZ8	**Down syndrome cell adhesion molecule homolog**	2111	−
P28023	**Dynactin subunit 1**	3064	−
O35964	**Endophilin-A2**	27839	+
P24942	**Excitatory amino acid transporter 1**	27055	−
Q3T1K5	**F-actin-capping protein subunit alpha-2**	8085	+
P04937	**Fibronectin**	2559	+
P04906	**Glutathione S-transferase P**	9312	+
D3ZZL9	**GRIP and coiled-coil domain-containing protein 2**	3734	+
P0C0K5	**Hepatitis A virus cellular receptor 2 homolog**	5942	+
Q5RKG2	**Host cell factor 2**	3844	−
Q99NA5	**Isocitrate dehydrogenase [NAD] subunit alpha, mitochondrial**	3615	−
Q68FX0	**Isocitrate dehydrogenase [NAD] subunit beta, mitochondrial**	5466	−
Q5XIA9	**Kelch domain-containing protein 8B**	5129	+
Q6IG00	**Keratin, type II cytoskeletal 4**	3867	+
Q6P6Q2	**Keratin, type II cytoskeletal 5**	3867	+
Q4FZU2	**Keratin, type II cytoskeletal 6A**	3867	+
Q6IG12	**Keratin, type II cytoskeletal 7**	3867	+
Q6IG05	**Keratin, type II cytoskeletal 75**	3867	+
Q10758	**Keratin, type II cytoskeletal 8**	3867	+
Q9QYU4	**Ketimine reductase mu-crystallin**	12012	+
Q91V33	**KH domain-containing, RNA-binding, signal transduction-associated protein 1**	3741	−
Q2PQA9	**Kinesin−1 heavy chain**	5374	+
Q4KLL9	**Kinesin-like protein KIF18B**	7570	+

Q6P7A9	**Lysosomal alpha-glucosidase**	12166	+
A1A5P9	**Melanoma-associated antigen E1**	3335	+
P63086	**Mitogen-activated protein kinase 1**	5624	+
Q03626	**Murinoglobulin−1**	4112	−
Q6IE52	**Murinoglobulin-2**	2849	−
Q5BK63	**NADH dehydrogenase [ubiquinone] 1 alpha subcomplex subunit 9, mitochondrial**	11676	−
A1L1I3	**Numb-like protein**	3366	+
O88488	**Phosphatidylinositol phosphatase PTPRQ**	4821	−
P11505	**Plasma membrane calcium-transporting ATPase 1**	9453	−
P11506	**Plasma membrane calcium-transporting ATPase 2**	4977	−
Q64568	**Plasma membrane calcium-transporting ATPase 3**	3948	−
Q64542	**Plasma membrane calcium-transporting ATPase 4**	2544	−
O35264	**Platelet-activating factor acetylhydrolase IB subunit beta**	10420	−
P30427	**Plectin**	2065	+
A1A5S1	**Pre-mRNA-processing factor 6**	3793	−
Q4KLM6	**Prolyl 3-hydroxylase 2**	7553	+
P05696	**Protein kinase C alpha type**	2389	+
Q9Z0W5	**Protein kinase C and casein kinase substrate in neurons protein 1**	11298	+
P68403	**Protein kinase C beta type**	2389	+
Q62919	**Protein kinase C-binding protein NELL1**	3143	−
Q8R508	**Protocadherin Fat 3**	2195	−
Q63713	**Ras GTPase-activating protein 2**	2387	+
Q64604	**Receptor-type tyrosine-protein phosphatase F**	2171	−
Q5XXR3	**Rho guanine nucleotide exchange factor 6**	4257	+
Q5PQK1	**Septin−10**	1680	−
B3GNI6	**Septin−11**	9876	−
Q9JJM9	**Septin-5**	11653	+
B0BNF1	**Septin-8**	1993	−
Q9R011	**Serine/threonine-protein kinase PLK3**	12021	+
P36876	**Serine/threonine-protein phosphatase 2A 55 kDa regulatory subunit B alpha isoform**	5542	−
O35412	**Signal-induced proliferation-associated 1-like protein 1**	6277	−
Q07116	**Sulfite oxidase, mitochondrial**	7611	+
Q5CD77	**TBC1 domain family member 14**	17646	+
Q6P502	**T-complex protein 1 subunit gamma**	34657	+
P37805	**Transgelin-3**	16870	+
F1LNJ2	**U5 small nuclear ribonucleoprotein 200 kDa helicase**	4944	+

D3ZJ96	**Ubiquitin carboxyl-terminal hydrolase 28**	3014	+
B2RYG6	**Ubiquitin thioesterase OTUB1**	8765	−
Q5U300	**Ubiquitin-like modifier-activating enzyme 1**	7179	+
D3ZYQ8	**Uridine-cytidine kinase**	2997	−
P31000	**Vimentin**	3867	+
P25286	**V-type proton ATPase 116 kDa subunit a isoform 1**	2497	−

[a]Accession ID according to Uniport.org database. Positive and negative values of fold change indicate up- and down-regulated proteins, respectively. Signs of + or – indicates exclusive expression in Pb group and in control group, respectively. Results of the comparison between Pb group and control group.

© 2020 by the authors. Licensee MDPI, Basel, Switzerland. This article is an open access article distributed under the terms and conditions of the Creative Commons Attribution (CC BY) license (http://creativecommons.org/licenses/by/4.0/).
